# Transcriptome Analysis of the Hepatopancreas in the Pacific White Shrimp (*Litopenaeus vannamei*) under Acute Ammonia Stress

**DOI:** 10.1371/journal.pone.0164396

**Published:** 2016-10-19

**Authors:** Xia Lu, Jie Kong, Sheng Luan, Ping Dai, Xianhong Meng, Baoxiang Cao, Kun Luo

**Affiliations:** 1 Key Laboratory of Sustainable Utilization of Marine Fisheries Resources, Ministry of Agriculture, Yellow Sea Fisheries Research Institute, Chinese Academy of Fishery Sciences, Qingdao 266071, China; 2 Laboratory for Marine Fisheries Science and Food Production Processes, Qingdao National Laboratory for Marine Science and Technology, Qingdao 266071, China; Shanghai Ocean University, CHINA

## Abstract

In the practical farming of *Litopenaeus vannamei*, the intensive culture system and environmental pollution usually results in a high concentration of ammonia, which usually brings large detrimental effects to shrimp, such as increasing the susceptibility to pathogens, reducing growth, decreasing osmoregulatory capacity, increasing the molting frequency, and even causing high mortality. However, little information is available on the molecular mechanisms of the detrimental effects of ammonia stress in shrimp. In this study, we performed comparative transcriptome analysis between ammonia-challenged and control groups from the same family of *L*. *vannamei* to identify the key genes and pathways response to ammonia stress. The comparative transcriptome analysis identified 136 significantly differentially expressed genes that have high homologies with the known proteins in aquatic species, among which 94 genes are reported potentially related to immune function, and the rest of the genes are involved in apoptosis, growth, molting, and osmoregulation. Fourteen GO terms and 6 KEGG pathways were identified to be significantly changed by ammonia stress. In these GO terms, 13 genes have been studied in aquatic species, and 11 of them were reported potentially involved in immune defense and two genes were related to molting. In the significantly changed KEGG pathways, all the 7 significantly changed genes have been reported in shrimp, and four of them were potentially involved in immune defense and the other three were related to molting, defending toxicity, and osmoregulation, respectively. In addition, majority of the significantly changed genes involved in nitrogen metabolisms that play an important role in reducing ammonia toxicity failed to perform the protection function. The present results have supplied molecular level support for the previous founding of the detrimental effects of ammonia stress in shrimp, which is a prerequisite for better understanding the molecular mechanism of the immunosuppression from ammonia stress.

## Introduction

The Pacific white shrimp, *Litopenaeus vannamei*, is one of the most important farmed penaeid shrimp species in the world, which currently provides about 52% of the total penaeid shrimp production in the world [1, 2]. In order to pursue more production value, semi-intensive and intensive culture systems are usually used for *L*. *vannamei* culture in many countries. However, these culture behaviors often result in degradation of the culture water from waste products of the shrimps and uneaten food. Combining with the variable and deteriorated aquaculture environments, the toxicity from the degraded culture water factors is playing an important role in the high mortality of shrimp, which has significantly affected the yield and quality of *L*. *vannamei* [3–6].

Among the degraded water factors, high concentration of ammonia is the commonest toxic factor to shrimp, which has been detected lethal effects on the penaeid shrimps, including *L*. *vannamei* [7], *Fenneropenaeus chinensis* [8], *Penaeus monodon* [9], *Farfantepenaeus paulensis* [10], *P*. *penicillatus* [11], *P*. *semisulcatus* [12], and *Metapenaeus ensis* [13]. The ammonia is usually present in ionized (NH_4_^+^) and un-ionized (NH_3_) state in water [7], but NH_3_ is the toxic form due to its ability to diffuse across cell membranes. High water temperature, high pH, and low salinity could increase the toxicity of ammonia to shrimp [14–21], because they increase the relative proportion of NH_3_ in water. Many studies have been performed to detect toxicity of ammonia to shrimp, and they stated the high concentration of ammonia harm shrimps in several ways. High concentration of ammonia could reduce growth rates, increase molting frequency, and even cause high mortality [22–24]. The osmoregulatory capacity of the shrimp decreases along with the ammonia concentration and exposure time increasing [25]. In addition, ammonia could damage the gills and hepatopancreas of shrimp, as well as reduces the ability of haemolymph to transport oxygen while increasing oxygen consumption [26, 27]. Importantly, it has been revealed that increased ammonia in the water could inhibit the immune system and increase the susceptibility of shrimp to pathogens [28, 29]. Liu and Chen [29] reported that mortality of *Vibrio*-injected shrimp held in ammonia was significantly higher than the *Vibrio*-injected and control groups after 48–168 h, and the mortality increased directly with ammonia concentration and expose time. The same situation also has been detected in shellfish and fish, such as abalone [30] and rainbow trout [31]. Unfortunately, the enlarged scope of human activities and the deepen degree of impact, as well as the changeable climate and global warming, jointly make the aquatic environments worse and the water quality factors more complex and out of control. The frequent outbreak of high mortality in the shrimp farms might be related to the enhanced toxicity of the ammonia from other multiple factors (such as higher temperature and low salinity by frequent rains in summer).

However, the molecular mechanisms of the detrimental effects of ammonia stress are poorly understood. Considering the inevitability of the complicated, fickle, and deteriorated aquaculture environments and the grievous harm of ammonia in shrimp aquaculture, it is vital to understand the molecular response mechanism to ammonia stress for seeking possibilities of culturing shrimp that efficiently tolerate ammonia stress and resist the pathogens, which might be an alternative method to reduce mortality and infectious diseases. To study the changes of gene expression and metabolic pathways under ammonia stress is a priority for understanding of the molecular response mechanism in the non-model species with rare genetic resources. Along with the recent development in next-generation sequencing (NGS) technologies, whole-transcriptome shotgun sequencing, also known as RNA sequencing (RNA-seq), is often used to capture and annotate the transcriptome for understanding the molecular mechanism of a specific physiological process, which could detect nearly all of the genes and pathways involved in the corresponding physiological function with high sensitivity [1, 32–34], which has been widely applied to the stress studies in the aquatic animals such as shrimp [35] and crab [36].

The previous study reported that high ammonia could heavily damage the hepatopancreas of shrimp [37]. Consequently, in the present study, we identified the genes and pathways respond to ammonia stress in the hepatpancreas of *L*. *vannamei* using the efficient and high-throughput method of RNA-Seq technology. The comparative transcriptome analysis was performed between an ammonia-challenged group and a non-challenged (control) group from the same family basing on the Illumina HiSeq 2500 platform. This study represents the first investigation of the molecular response to ammonia stress in shrimp at whole transcriptome level.

## Materials and Methods

### Shrimps and ammonia challenge experiment

Forty seven families of *L*. *vannamei* were produced at the Mariculture Genetic Breeding Center of the Chinese Ministry of Agriculture (Qingdao, China). In order to know how much of the trait of ammonia tolerance in *L*. *vannamei* is genetically involved based on the additive genetic effect model, we estimated the heritability of ammonia tolerance with these families [38]. Before the heritability estimation, we carried out a pre-experiment to detect the half-lethal time under our experiment condition with the ammonia doses reported by Sun et al. [39], because the toxicity of ammonia is largely depend on temperature, pH, salinity, and body weight, etc. In order to reduce the impacts from body weight, a half-lethal dose (~ 62.23mg/L) with shorter lethal time (120 h) was selected for the heritability estimation. Interestingly, the individuals from a family died significantly earlier than the other families according to the individual death time that was used as the evaluation index, so we defined this family as an “ammonia-sensitive” family. Under the situation that we were not very sure for the ammonia tolerance of the other families, we think the individuals from this “ammonia-sensitive” family would have more significant changes when exposed to ammonia stress, which might provide a better material for investigating the detrimental effects from ammonia stress. In addition, the control group from the same family could efficiently minimize the false positives that result from difference of genetic background. Therefore, the individuals from this “ammonia-sensitive” family were used for identification of significantly changed genes and pathways response to ammonia stress. Considering the experiment family was selected basing on its performance under the concentration of ~ 62.23mg/L, so we still used this dose level for the present study.

Before the challenge experiment, a total of 60 shrimp were randomly selected from the “ammonia-sensitive” family and equally divided into two groups and acclimated for one week at the Mariculture Genetic Breeding Center of the Chinese Ministry of Agriculture. During the period of acclimation, the two groups were feeding a commercial diet four times per day, which containing 12% moisture, 44% crude protein and 16% crude ash. In addition, 50% of their water was exchanged every day to maintain the ammonia concentration at normal level (less than 0.2 mg/L). During the acclimation, the holding water conditions were as follows: salinity at 30‰, pH at 7.9 ± 0.1, temperature at 27 ± 0.5°C, and oxygen higher than 6 mg/L. After the acclimation, one group was treated as the control (LV_C), and the other group (LC_E) was challenged with high-concentration ammonia (~ 62.23 mg/L) according to our previous study [38]. The temperature was 27 ± 0.5°C, pH was 7.9 ± 0.1, and salinity was 30‰. The ammonia dose used in the present study would lead to an acute stress on shrimp. We are more interested in the changes of gene expression and pathways at the early stage of acute ammonia stress. So when the shrimps were exposed to high-concentration ammonia for 10 hours, the hepatopancreas of ten individuals from each group were separately dissected and frozen immediately in liquid nitrogen, and after that they were stored at -80°C until RNA extraction, which was completed within one week.

### RNA isolation and Quality control

For each group, six individuals were subjected to RNA extraction. The total RNA was extracted separately from the hepatopancreas of *L*. *vannamei* using Trizol Reagent (Qiagen, Hilden, Germany) following with the manufacturer’s protocol. In order to make the total RNA samples have high quality, great care has been taken in each step. The genomic DNA was cleaned from RNA with RNase free DNas I (Takara, Japan), and the degradation and contamination of the RNA was detected by 1.5% agarose gels. After that, the purity, concentration, and integrity of the RNA were checked using NanoPhotometer spectrophotometer (Implen, GER), Qubit RNA Assay Kit in Qubit 2.0 Fluorometer (Life Technologies, CA, USA), and RNA Nano 6000 Assay Kit of the Bioanalyzer 2100 system (Agilent Technologies, CA, USA), respectively, which also has been described by Huang et al. [40]. According to the checking result, all of the RNA samples have high-quality (OD_260/280_ = 2.0–2.2, OD_260/230_ ≧ 2.0, RIN ≧ 8.0, and 28S:18S ≧ 1.0). The present samples were from the same families in the present study, which could efficiently minimize the interference from difference genetic background and the false positive. So RNA from three individuals was made a pool to construct a sequencing library, by which two biological replicates were used for each group in the present study. A total amount of 3 μg RNA with high quality was used for each library construction.

### Library preparation and Illumina sequencing

The library was constructed using Illumina TruSeq^TM^ RNA Sample Peparation Kit (Illumina, USA) according to the manufacture’s recommendation. First, poly-T oligo-attached magnetic beads were used to purify the mRNA from the pooled RNA, after that the purified mRNA was cleaved into small fragments by divalent cations in the fragmentation buffer under increased temperature. Then, first-strand cDNA was synthetized with the cleaved RNA fragments with M-MuLV Reverse Transcripase (RNase H) and random hexamer primer. The second-strand cDNA was synthetized with RNase H and DNA polymerase I. Adapters were ligated to the synthetized cDNA fragments after an end repair step, and the products with 200–300 bp were selected on 2% ultra-agarose. These cDNA fragments were performed PCR amplification for 15 cycles with DNA polymerase (QIAGEN, German) for library construction. The cDNA libraries were then sequenced on the Illumina HiSeq2500 sequencing platform and 125 bp paired-end raw reads were generated.

### De novo transcriptome assembly and gene function annotation

The adapter sequences, low quality reads, and reads with ploy-N were removed from the raw reads, and then the high quality reads were used for the downstream analysis. The obtained clean reads were then randomly clipped into overlapping K-mers with default K = 25 for assembly with the Trinity software [41]. The non-redundant sequences were subjected to BLAST searches and annotations against the Swiss-Prot (http://www.ebi.ac.uk/uniprot/)) and NCBI (http://www.ncbi.nlm.nih.gov/) non-redundant protein (Nr) and non-redundant nucleotide (Nt) using BlastX algorithm with an E-value cut-off of 10^−10^. After that GO (Gene Ontology terms, http://www.geneontology.org/), KOG/COG (Clusters of Orthologous Groups of proteins, http://www.ncbi.nlm.nih.gov/COG/)) annotation, and KEGG classification (http://www.genome.jp/kegg/) were analyzed with Blast2GO, BlastX 2.2.24+, and BlastX/BlastP 2.2.24+ software, respectively. If the annotation result from the different databases is conflicted, the priority order of alignments for the databases was Nr, Nt, KEGG, Swiss-Prot, GO, and COG.

### Differential expression and cluster analysis

The samples of the present study had biological replicates; therefore, the DEGseq R package (2010) was used to perform a differential expression analysis for the two groups (LV_E and LV_C), which provides statistical routines for determining differentially expressed genes (DEGs) using a model basing on the negative binomial distribution. Benjaminiand Hochberg’s approach was used to adjust the resulting *P* value (q-value) for monitoring false discovery rate [42]. The genes with a q-value < 0.05 were assigned as significantly differential expression. In addition, a cluster analysis was performed to identify DEGs between LV_E and LV_C using an R package of *pheatmap*. The genes were clustered according to the relative expression level (log2 (ratios)) between the two groups.

### Enrichment analysis of GO and KEGG

A functional-enrichment analysis was performed to identify the DEGs that were significantly enriched in GO terms (with q-value < 0.05) relative to the whole-transcriptome background, which was implemented by Goatools (https://github.com/tanghaibao/Goatools). The KEGG pathway is vital for understanding the functions and utilities of the biological system from molecular-level information [43], so enrichment analysis also was performed to identify the DEGs that were significantly enriched in KEGG pathways (with q-value < 0.05) relative to the whole-transcriptome background with the KOBAS software [44].

### Identification of key genes response to acute ammonia stress

It has been revealed that increased ammonia in the water could bring heavy detrimental effects to shrimp, such as inhibit the immune system and increase the susceptibility of shrimp to pathogens, reduce growth, and even cause high mortality, which was very important for the shrimp farming, so identification of ammonia-stress response genes was carried out from the DEGs in the BLASTX alignment results with Nr, Nt, KEGG, Swiss-Prot, PFAM, GO and KOG databases. The Glycosylphosphatidylinositol (GPI) anchored proteins were detected using the GPI Prediction Server (http://mendel.imp.ac.at/sat/gpi/gpi_server.html) and then removed from the results [45, 46]. The DEGs enriched to the significantly changed GO terms and KEGG pathways (q value < 0.05) under acute ammonia stress were selected and identified as genes potentially response to ammonia stress. In addition, the pathways that might reduce ammonia toxicity at cellular and molecular levels was also important for understanding the molecular mechanisms of detrimental effects of ammonia stress, so we also play attention on DEGs involved in nitrogen metabolisms that reduce the toxicity of ammonia. The unigene with maximum E-value was selected as the representative, when several unique transcripts were assigned to the same reference gene.

### Gene expression validation

For validation of the Illumina sequencing data, twelve differentially expressed genes were selected to be quantified by real-time PCR with the same six separate RNA samples used for the Illumina sequencing in each group. Their primers were designed using the Primer Premier 5 software (Premier Biosoft International) according to Illumina sequencing data. The details of the primers are displayed in S1 Table. The 18S gene of *L*. *vannamei* was selected as an internal control to normalize the expression level; its primers (F: TATACGCTAGTGGAGCTGGAA, R: GGGGAGGTAGTGACGAAAAAT, and Ta: 56°C) referenced in the previous study by Zhang et al. [47]. After the mRNA was reverse transcribed into cDNA, real time PCR was performed in an ABI 7900 HT Sequence Detection System (ABI, USA) and all of the samples were performed in triplicate. RT-PCR was carried out in a total volume of 10 μL, containing 5 μL of 2× SYBR Green PCR buffer, 0.5 μL of each primer (10 μM), 5 ng of cDNA and Milli-Q water added to reach a final volume of 10 μL. The PCR cycling parameters as follows: 50°C for 2 min, 95°C for 10 min, followed by 40 cycles of 95°C for 15 s and 60°C for 1 min. In order to ensure that only one PCR product was amplified, a dissociation curve analysis was performed for the products at the end of each PCR reaction. The data were analyzed using comparative CT method (2^-ΔΔ^ CT method) for the expression level of the genes.

## Results

### Transcriptome sequencing and assembly

Illumina sequencing totally generated 239,706,580 raw reads, which were deposited in the Short Read Archive (SRA) of the National Center for Biotechnology Information (NCBI) with the accession number of SRP062191. The mean values of the Q20 percentage and Q30 percentage are 96.47% and 92.97%, respectively, and the error rate is 0.03%. After removing the adapter sequences, ambiguous nucleotides and low-quality sequences, a total of 233,267,140 clean reads were generated through Illumina sequencing, containing 116,398,608 reads for the two LV_E libraries and 116,868,532 reads for the two LV_C libraries. A total of 29.14 G clean bases were generated from the clean reads, and the average percentage of GC content for the clean reads was 48.32%.

The clean bases were performed *de novo* assembly using Trinity software and assembled into 94,627 transcripts with a total length of 93,657,683 nucleotides (Table 1). The length of the transcripts ranged from 201 bp to 38,359 bp with an average length of 990 bp and N50 of 38,395. These transcripts were subsequently assembled into 78,636 unigenes that ranged from 201 bp to 38,359 bp with an average length of 797 bp and N50 of 1,597. N50 length is defined as the contig length L for which 50% of all bases in the sequences is in contigs of length less than L. The total length of the unigenes is 62,678,462 bp, which covered 66.92% of the length of transcripts.

**Table 1:** Summary of *de novo* assembly results of the transcriptome in hepatopancreas of *Litopenaeus vannamei*.

	Min length	Mean length	Median length	Max length	N50	N90	Total nucleotides
**Transcripts**	201	990	413	38,359	38,359	328	93,657,683
**Unigenes**	201	797	365	38,359	1,597	281	62,678,462

### Functional annotation of unigenes

After removing the low-quality and short-length sequences, an annotation analysis was performed on the remaining 78,636 non-redundant unigenes by matching sequences against the public databases, and the annotation results are summarized in Table 2. The highest percentage of unigenes was annotated in the GO database, accounting for 19,099 (24.28%) of all unigenes, followed by 15,394 (19.57%) annotated in the Nr database and 12,311 (15.65%) matched to the SwissProt database. There were 1,743 (2.21%) unigenes annotated in all of the databases, and 22,679 (28.84%) unigenes were annotated in at least one database. The E-values and similarity distributions of the best hits in the Nr database are shown in S1 Fig Among the unigenes successfully annotated in the Nr database, strong homology (E-value less than 10^−30^) was observed in 10,360 (67.3%) unigenes and 12,715 (82.6%) matched sequences had high homology with an E-value < 10^−15^ (S1A Fig). There were 10422 (67.9%) unigenes aligned with a similarity index > 60% according to the similarity distribution (S1B Fig).

**Table 2:** Summary of the annotations of the unigenes under ammonia stress in *Litopenaeus vannamei*.

All Unigenes	Number of Unigenes	Percentage (%)
**Annotated in Nr**	15,394	19.57
**Annotated in Nt**	3,101	3.94
**Annotated in KEGG**	6,856	8.71
**Annotated in SwissProt**	12,311	15.65
**Annotated in GO**	19,099	24.28
**Annotated in KOG**	9,483	12.05
**Annotated in all Databases**	1,743	2.21
**Annotated in at least one Database**	22,679	28.84
**Total Unigenes**	78,636	100

**Fig 1:**
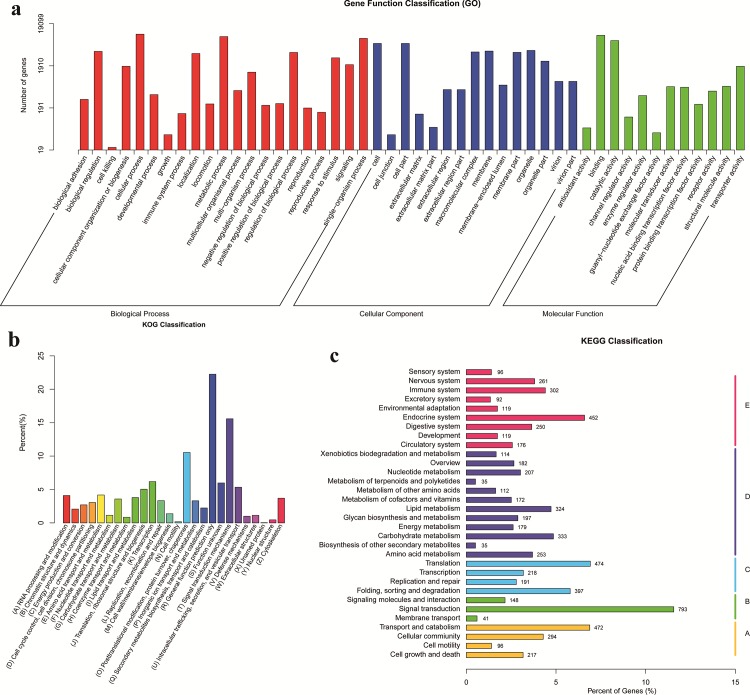
(a) GO categorization of non-redundant unigenes from the *de novo* assembly of LV_E and LV_C reads, and each annotated unigene was assigned at least one GO term. (b) KOG annotation of putative proteins. (c) KEGG classification of non-redundant unigenes.

According to GO terms, a total of 19,099 unigenes were classified into three major functional categories, containing biological progress, cellular component, and molecular function (Fig 1A). Among these annotated unigenes, 46.22% are classified to the category of biological process, 32.90% to cellular component, and 20.88% to molecular function. The category of biological process was grouped to 21 subcategories, in which the major subcategories were cellular process (21.43%) and metabolic process (18.76%). The category of cellular component contained 15 subcategories, among which cell (18.15%) and cell part (18.14%) were the dominant subcategories. In the category of molecular function, the major subcategories were binding (44.58%) and catalytic activity (33.36%).

A total of 9,483 unigenes were categorized into 26 KOG classifications, among which the (R) General Function Prediction Only (22.24%) was the dominant subcategory, followed by (T) Signal Transduction Mechanisms (15.59%), (O) Posttranslational Modification, Protein Turnover, and Chaperones (10.55%) (Fig 1B). In contrast, the subcategories of (N) Cell motility (0.16%) and (Y) Nuclear structure (0.40%) covered fewer unigenes.

The biological pathways for the unigenes were searched using KEGG database, by which 6,856 unigenes were assigned to 5 special KEGG pathways, including Metabolism (31.26%, D), Organismal Systems (27.23%, E), Genetic Information Processing (18.67%, C), Cellular Processes (15.74%, A), and Environmental Information Processing (14.32%, B) (Fig 1C). The annotated unigenes are involved in 260 different pathways. Among all the KEGG classification, the largest number of unigenes (793) is assigned to the pathway of signal transduction, accounting for 80.75% of the total annotated unigenes in the category of environmental information processing, followed by translation pathway (474 unigenes), transport and catabolism pathway (472 unigenes), and endocrine system pathway (452 unigenes) in the categories of genetic information processing, cellular processes, and organismal systems, respectively. Additionally, there are a total of 302 unigenes identified in immune system pathway.

### Differential expressed genes

**Fig 2:**
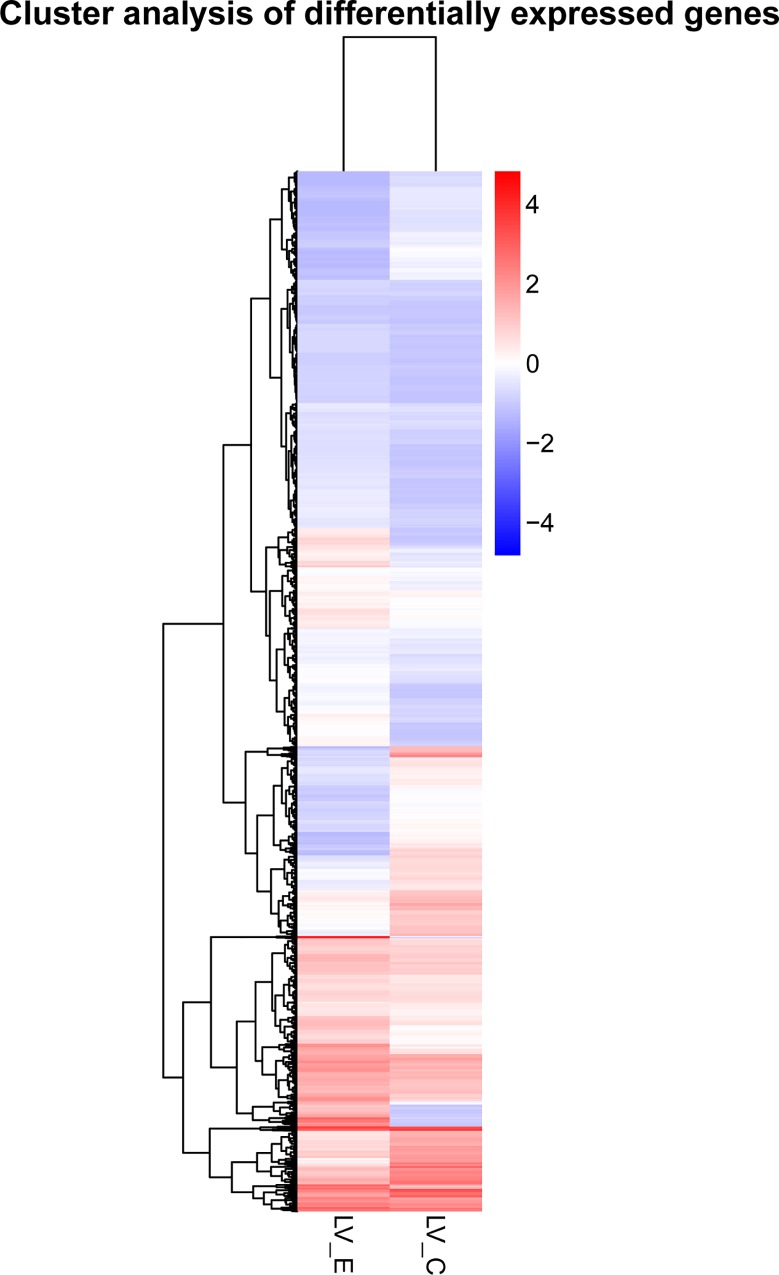
The hierarchical clustering for the differentially expressed genes between LV_E and LV_C. The red color shows the high expression, and the blue color represents the down expression. The color from red to blue represents the log10 (FPKM+1) from large to small.

To identify differentially expressed genes response to acute ammonia stress, comparative transcriptome analysis was performed between ammonia-stress group and control group with the threshold of q value < 0.05. A total of 3,145 significantly differentially expressed unigenes were observed between LV_E and LV_C, containing 1,097 down-regulated genes and 2048 up-regulated genes. The global expression of the DEGs were further estimated by a hierarchical cluster analysis according to the relative expression level (log2 (ratios)) between LV_E and LV_C. The hierarchical clustering of the DEGs provided an intuitive way to display the clustering patterns of the DEGs between the two groups, which showed that the expression pattern of the DEGs in LV_E was distinguishable from that in LV_C (Fig 2).

For the 3,145 differentially expressed unigenes, 1826 (58.06%) genes were annotated in the public databases, and their annotation details are presented in additional S2 Table. Many of the candidate genes that respond to ammonia stress are related to immune defense, apoptosis, growth, osmoregulation, and molting (S2 Table). Alignments of annotated unigenes with the sequences of known proteins revealed 136 unigenes with strong homologies to known genes in the aquatic species, including 44 genes in *L*. *vannamei*, 25 in *P*. *monodon*, 18 in *Marsupenaeus japonicas*, 12 in *F*. *chinensis*, and 14 in *Crassostrea gigas*, etc. (S3 Table). We pay more attention for these 136 genes, so verify their function in aquatic species by searching and reading their published articles, and supplied their reported function and reference information in S3 Table. The meaningful finding is that most of these genes (94, 69.1%) are related to immune defense function, and the rest of the genes play an important role in osmoregulation, molting, growth, gonad development, and response to stress (S3 Table). The significantly changed unigenes that have high homology to known genes in *L*. *vannamei* is listed in Table 3 (more information in S3 Table). As expected, majority of these genes (35, 79.5%) potentially involved in the immune function in *L*. *vannamei*, such as penaeidin, putative antimicrobial peptide, prophenoloxidase, prophenoloxidase activating enzyme, alpha-2-macroglobulin, and C type lectin, etc.

**Table 3:** List of the candidate genes have homologies with known proteins in *Litopenaeus vannamei* under ammonia stress.

Annotation	FC*	Function	Annotation	FC*	Function
crustin-like protein	-3.65	Immune Defense	single WAP domain protein	-1.39	Immune Defense
ENSANGP00000021035-like	-1.38	Immune Defense	single VWC domain protein 4	3.11	Immune Defense
single VWC domain protein 5	-2.43	Immune Defense	transcription factor ATF-b	1.02	Immune Defense
putative antimicrobial peptide	-3.23	Immune Defense	integrin beta subunit	0.85	Immune Defense
retinoblastoma family-like protein	-1.25	Immune Defense	eukaryotic initiation factor 4A	0.74	Gonad development
sterile-alpha and armadillo motif containing protein	2.04	Immune Defense	prophenoloxidase activating enzyme 2	-1.87	Immune Defense
lactate dehydrogenase	2.79	Response to stress	HMGBb	-0.83	Immune Defense
chitinase 1 precursor	3.86	Immune Defense	cyclin A	2.43	Ovarian development
elongation factor 2	0.97	Response to stress	peritrophin	2.44	Immune Defense
serine proteinase inhibitor	2.81	Immune Defense	hemolin-like protein	2.43	Immune Defense
prophenoloxidase-2	-3.66	Immune Defense	ecdysteroid receptor E75	1.00	Molting
X-box binding protein 1 splicing form	1.21	Immune Defense	prophenoloxidase activating factor	-1.74	Immune Defense
alpha-2-macroglobulin	-3.03	Immune Defense	C type lectin containing domain protein	-2.33	Immune Defense
Penaeidin-2a	-3.60	Immune Defense	Penaeidin-3d	-2.26	Immune Defense
mas	-2.93	Immune Defense	inositol-requiring enzyme-1	1.05	Response to stress
kruppel-like factor	1.48	Immune Defense	cytochrome P450	-0.94	Immune Defense
Penaeidin-4a	-3.24	Immune Defense	prophenoloxidase activating enzyme	-1.87	Immune Defense
pellino	1.10	Immune Defense	cyclin B	2.15	Ovarian development
chicadae/profilin	2.73	Immune Defense	cytochrome c	0.77	Apoptosis
Kazal-type proteinase inhibitor	-2.10	Immune Defense	integrin	0.85	Immune Defense
triose-phosphate isomerase	-0.70	Immune Defense	glucose transporter 1	-1.20	Immune Defense
alpha glucosidase	2.38	Growth	phosphoenolpyruvate carboxykinase	1.26	Growth

*, LV_E vs. LV_C log2FoldChange.

### Functional enrichment analysis of GO and KEGG pathways

For the DEGs between LV_E and LV_C, the main molecular functions they exercise were identified by GO and KEGG pathway enrichment analysis. A total of 14 significantly changed GO terms (q value < 0.05) were obtained in the differentially expressed genes (S2 Fig). These GO terms were classified into three categories, containing four, two, and eight terms for the categories of biological progress, cellular component, and molecular functions, respectively. Excepting for the replicates of unigenes that were enriched to different GO terms and after eliminating the different unigenes that assigned to the same reference gene, 87 genes were observed in the 14 significant changed GO terms (details in S4 Table). According to their function annotation, most of these genes are potentially related with immune function, such as bacterial extracellular solute-binding proteins, peritrophin, chitinase, C-type lectin, and G protein-coupled receptor, etc. Among these annotated unigenes, there are 13 unigenes have high homology to known proteins in the aquatic species (Table 4), most of which are potentially involved in immune function (S3 Table). They are assigned to 9 significant GO terms, including chitin metabolic process (GO:0006030), chitin binding (GO:0008061), amino sugar metabolic process (GO:0006040), glucosamine-containing compound metabolic process (GO:1901071), aminoglycan metabolic process (GO:0006022), carbohydrate derivative binding (GO:0097367), transmembrane signaling receptor activity (GO:0004888), receptor activity (GO:0004872), and signaling receptor activity (GO:0038023).

**Table 4:** List of the genes have homologies to known proteins of aquatic species in significant changed GO terms under ammonia stress in *Litopenaeus vannamei*.

Description	FC*	Function	Enriched GO terms
ENSANGP00000021035-like	-1.38	Immune Defense	• Amino sugar metabolic process • Chitin metabolic process • Glucosamine-containing compound metabolic process • Aminoglycan metabolic process • Chitin binding • Carbohydrate derivative binding
chitinase	-1.53	Molting and Immune Defense	
ovarian peritrophin	2.85	Immune Defense	
thrombospondin II	3.53	Immune Defense	
thrombospondin protein	3.42	Immune Defense	
olfactory ionotropic receptor IR93a	3.17	Olfactory signaling	• Transmembrane signaling receptor activity • Receptor activity • Signaling receptor activity
G protein-coupled receptor	-2.24	Immune Defense	
Penaeidin-2a	-3.60	Immune Defense	• Chitin binding • Carbohydrate derivative binding
Penaeidin-4a	-3.24	Immune Defense	
ecdysteroid receptor E75	1.00	Molting	• Receptor activity • Signaling receptor activity
cation-independent mannose-6-phosphate receptor	1.62	Immune Defense	
Penaeidin-3d	-2.26	Immune Defense	Carbohydrate derivative binding
integrin	0.85	Immune Defense	Receptor activity

*, LV_E vs. LV_C log2FoldChange.

**Fig 3:**
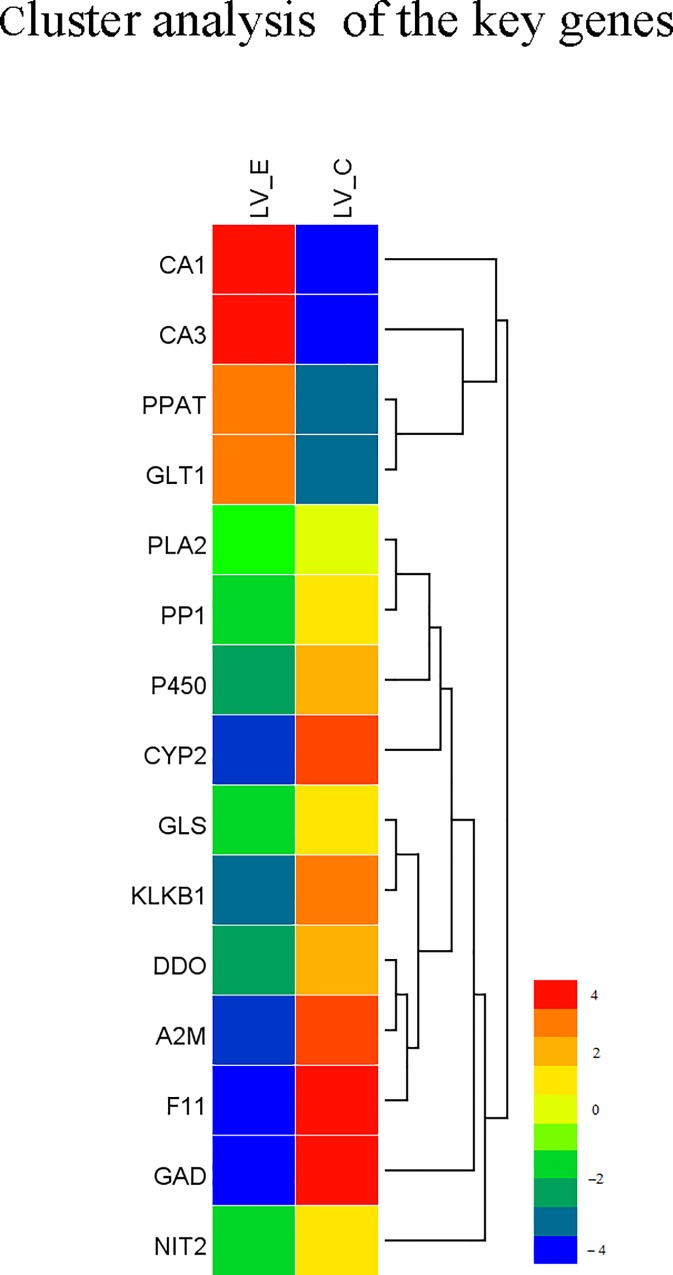
The hierarchical clustering for the key differentially expressed genes between LV_E and LV_C from functional enrichment analysis of GO and KEGG pathways. The red color shows the high expression, and the blue color represents the down expression. The color from red to blue represents the log10 (FPKM+1) from large to small.

**Fig 4:**
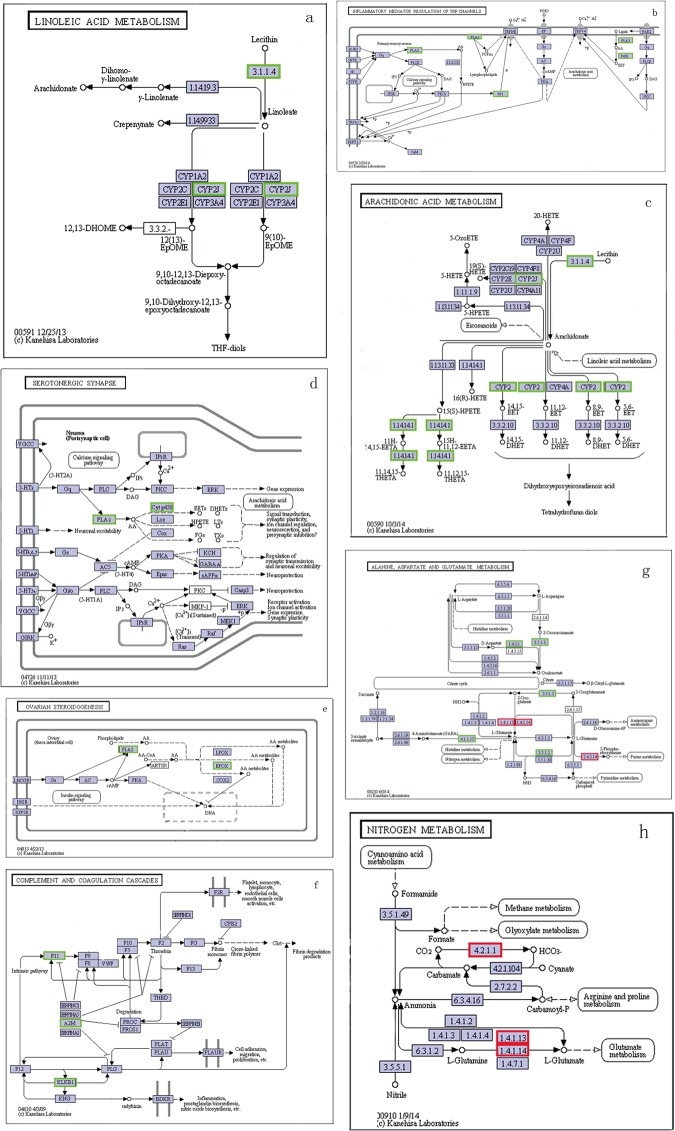
The six significant changed KEGG pathways and two nitrogen metabolism pathways: a, Linoleic acid metabolism (ko00591); b, Inflammatory mediator regulation of TRP channels (ko04750); c, Arachidonic acid metabolism (ko00590); d, Serotonergic synapse (ko04726); e, Ovarian steroidogenesis (ko04913); f, Complement and coagulation cascades (ko04610); g, nitrogen metabolism pathway (ko00910); h, alanine, aspartate and glutamate metabolism pathway (ko00250). The green frames represents the genes were down-regulated, and the red frames represents the genes were up-regulated.

In the enrichment analysis of KEGG pathway for the DEGs between the two groups, a total of 6 significantly changed KEGG pathways (q values < 0.05) were detected (S3 Fig). After eliminating the replicates of unigenes that were enriched to different KEGG pathways and the different unigenes that assigned to the same reference gene, 7 genes were identified from the 6 significantly changed KEGG pathways, and 8 genes were observed response to ammonia stress in nitrogen metabolism pathway (ko00910) and alanine, aspartate and glutamate metabolism pathway (ko00250) that could reduce the toxicity of ammonia (Table 5). The network diagram of these KEGG pathways showed that majority of the 15 genes were at the critical nodes of the pathways and were significantly down regulated by ammonia stress (Fig 3 and Fig 4). CYP2J enriched in Linoleic acid metabolism (ko00591) and Arachidonic acid metabolism (ko00590) was significantly down regulated (Fig 4A and 4C); PP1 enriched in Inflammatory mediator regulation of TRP channels (ko04750) was significantly down regulated (Fig 4B); P450 enriched in Inflammatory mediator regulation of TRP channels (ko04750) and Serotonergic synapse (ko04726) was significantly down regulated (Fig 4B and 4D); PLA2 enriched in Inflammatory mediator regulation of TRP channels (ko04750), Serotonergic synapse (ko04726), and Ovarian steroidogenesis (ko04913) was significantly down regulated (Fig 4B, 4D and 4E); F11, A2M, and KLKB1 enriched in Complement and coagulation cascades (ko04610) were significantly down regulated (Fig 4F); CA1 and CA3 enriched in alanine, aspartate and glutamate metabolism (ko00250) were significantly up regulated (Fig 4G); GLT1 enriched in alanine, aspartate and glutamate metabolism (ko00250) and nitrogen metabolism (ko00910) was significantly up regulated (Fig 4G and 4H); PPAT enriched in nitrogen metabolism pathway (ko00910) was significantly up regulated (Fig 4H), but the rest of the four genes enriched in this pathway were significantly down regulated (Fig 4H).

**Table 5:** List of the genes in significantly changed KEGG pathways under acute ammonia stress in *Litopenaeus vannamei*.

Gene name	FC*	Description	KO ID
P450	-2.82	Cytochrome P450	ko04750, ko04726
F11	-3.69	mas	ko04610
A2M	-4.58	alpha 2 macroglobulin	ko04610
CYP2J	-1.50	CYP2	ko00590, ko00591
PLA2	-0.72	cytosolic phospholipase A2	ko04750, ko04726, ko04913
PP1	-1.11	Serine/threonine-protein phosphatase	ko04750
KLKB1	-1.86	prophenoloxidase activating enzyme	ko04610
CA1	2.66	carbonic anhydrase 1	ko00250
CA3	3.14	carbonic anhydrase 3	ko00250
GLT1	1.93	glutamate synthase	ko00250, ko00910
PPAT	2.13	amidophosphoribosyl transferase	ko00910
DDO	-1.36	D-aspartate oxidase	ko00910
NIT2	-0.92	omega-amidase NIT2	ko00910
GAD	-2.16	glutamate decarboxylase	ko00910
GLS	-0.97	glutaminase kidney isoform	ko00910

*, LV_E vs. LV_C log2FoldChange.

### Validation of RNA-seq results by RT-PCR

**Fig 5:**
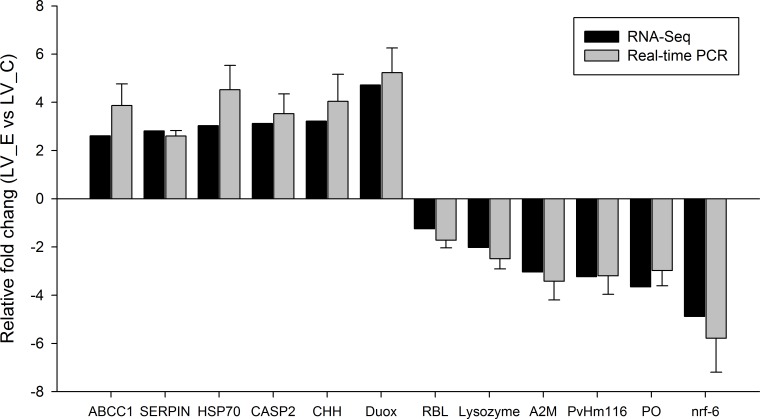
Validation results of RNA-seq profiles by real time PCR.

For the twelve differentially expressed genes that were selected for validation of the Illumina sequences by real-time PCR analysis, their quantitative results were all consistent with the results of the RNA-seq technology (Fig 5) and have high amplification efficiencies (E ranged from 0. 94 to 1.01). According to their annotation, most of the reanalyzed genes by real-time PCR were potentially involved in the immune functions, molting and apoptosis. These results confirm the reliability of RNA-seq and accuracy of the Trinity assembly, which not only verified the differential expression of the genes from the Illumina sequencing data but also validated the reliability of some ammonia responsive genes.

## Discussion

Increased ammonia in the water has heavy detrimental effects on shrimp, but little information was available for the molecular mechanism of the detrimental effects of ammonia stress to shrimp. This study is the first report on the key genes and metabolic pathways respond to the ammonia stress in shrimp by RNA-Seq technology, which provided much basic information for understanding the molecular mechanisms of the detrimental effects of ammonia stress in shrimp. According to the previous reports, ammonia stress could inhibit the immune system and increase the susceptibility of shrimp to pathogens [28, 29]. Interestingly, majority of the key genes response to ammonia stress are potentially involved in the function of immune defense.

In the present study, after eliminating the different unigenes that assigned to the same reference gene, 136 unigenes with strong homologies to known genes in the aquatic species were revealed response to acute ammonia stress, including 44 genes in *L*. *vannamei*, 25 in *P*. *monodon*, 18 in *M*. *japonicas*, 12 in *F*. *chinensis*, and 14 in *C*. *gigas*, etc. (S3 Table). The meaningful finding is that 69.1% of these genes are potentially related to immune defense function, and the rest of the genes play an important role in osmoregulation, molting, growth, gonad development, and response to stress (S3 Table), which supplied important molecular level evidence for the results reported in the preview studies [23–25, 28, 29]. As expected, majority of the genes (79.5%) that have strong homologies with known proteins in *L*. *vannamei* were potentially involved in the immune function, such as penaeidin, putative antimicrobial peptide, prophenoloxidase, prophenoloxidase activating enzyme, alpha-2-macroglobulin, peritrophin, and C type lectin, etc. (S3 Table). These candidate genes provide an important basis for screening the key genes response to ammonia stress.

In order to get the functional overview for the DEGs that were detected between LV_E and LV_C, we performed enrichment analysis of GO and KEGG pathways to identify the main molecular functions the DEGs involved, which would help us understand the potential molecular mechanism for the damage of ammonia stress. Thirteen genes have high homology with known proteins in the aquatic species were observed in the 14 significantly changed GO terms (q value < 0.05). As expected, 11 genes are potentially related with immune function, including ENSANGP00000021035-like, chitinase, ovarian peritrophin, thrombospondin, G protein-coupled receptor, penaeidin, mannose-6-phosphate receptor, and integrin. Wang et al. [48] have detected ENSANGP00000021035-like gene plays an important role in the antiviral immunity of *L*. *vannamei* by suppression subtractive hybridization (SSH) method. The chitinase is a multi-gene family, which plays important physiological roles in Crustacea, e.g. molting and defense against pathogen, and their innate immunity activity was reported in *L vannamei* [49]. Shrimp ovarian peritrophin (SOP) is proposed to play a role in the protection of spawned eggs, and the SOP in *F*. *merguiensis* displayed a chitinase activity and efficiently inhibited the growth of *Vibrio harveyi* and Staphylococcus aureus [50]. Pongsomboon S et al [51] have identified viral (white spot and yellow head viruses) and bacterial (*Vibrio harveyi*) in *P*. *monodon* with a cDNA microarray, and they revealed thrombospondin defend against pathogen infection. G protein-coupled receptor (GPCR) has immune function in mammals, and it was revealed that HP1R (a putative GPCR identified in *Procambarus clarkii*) was an important immune molecule which was required for red swamp crayfish to defend against bacterial infection [52]. In crustaceans, the synthesis of antimicrobial peptides or polypeptides induced upon injury is a major and important component of the humoral innate host defense, and penaeidins (penaeidin 2b, Penaeidin 3d, and Penaeidin 4a) are the important antimicrobial peptides in *L*. *vannamei* [53, 54]. It was reported that mannose-6-phosphate receptor involved in the immunological function in invertebrates [55]. Integrins are a family of adhesion receptors which regulate cell proliferation, differentiation, leukocyte migration, and complement receptor-dependent phagocytosis, and integrins β play an important role for the balanced activation of immune defense responses especially during the encounter of infections in *L*. *vannamei* [56, 57]. However, the expression of most of the above involved genes was significantly down regulated by ammonia stress, which would reduce the immune defense ability and increase the susceptibility to pathogens when the shrimp exposing to ammonia stress.

In addition, six KEGG pathways were significantly changed (q value < 0.05) by ammonia stress in the present study. The linoleic acid metabolism pathway (ko00591) and arachidonic acid metabolism pathway (ko00590) probably involved in growth. Kanazawa et al. [58] reported that linoleic acid as well as other fatty acids, including arachidonic acid, has an important positive impact on the growth of prawns [59]. However, the above mentioned two pathways both were significantly down-regulated, indicating that the ammonia stress reduce the growth rate of shrimp maybe by inhibiting these two pathways, which have provided molecular level evidence for the previous results reported by Chen and Kou [24]. Steroid hormones help control metabolism, inflammation, immune functions and salt and water balance, and they also influence sex characteristics and promote illness and injury prevention [1, 60–62]. In addition, Birukawa et al. [63] reported that steroid hormones were involved in the osmoregulation of cetaceans, which was consist with the previous investigation of the toxicity of ammonia in shrimp [25]. The other three significantly changed pathways (ko04726, ko04750, and ko04610) had an important impact on immune functions according to the previous study [64], and all of them were significantly down-regulated by ammonia stress.

Seven genes were identified from the 6 significantly changed KEGG pathways. All of the seven genes have been well studied in the aquatic species, among which five genes were potentially related to immune defense function, including alpha 2 macroglobulin (A2M), mas (F11), prophenoloxidase (KLKB1), Cytochrome P450 (P450), and Serine/threonine-protein phosphatase (PP1) (Table 5). The network diagram of the six KEGG pathways showed that the above five genes were at the critical nodes of the pathways (Fig 4), indicating they play important roles in these metabolic pathways. A2M is an evolutionarily conserved element of the innate immune system and a non-specific protease inhibitor involved in host defense, and it has been revealed that A2M is relative to immunity in *L*. *vannamei* [65]. The F11 gene (GenBank: AFW98990.1) was reported to play a role in immunity [1]. Recent studies revealed the importance of KLKB1 in shrimp immune response, particularly towards protect animals from the microbial pathogens [66]. Aquatic animals metabolize foreign toxicity of chemicals mainly by oxidation, reduction, hydrolysis and conjugation reactions catalyzed by various enzymes, and the metabolic activation is primarily catalyzed by the cytochrome P450-dependent oxygenase system in the endoplasmic reticulum [67]. Renault et al. [68] revealed PP1 potentially responds to virus infection in Pacific cupped oyster by SSH method and real time PCR. However, the expression of all the seven genes was significantly down regulated by ammonia stress, indicating that ammonia stress might reduce immune system activity by inhibiting the expression of the key genes in these pathways, which also supplied molecular level support for the finding in the previous studies [28, 29]. The genes have high homologies with known proteins in aquatic species and enriched to the significantly changed GO terms and KEGG pathways are selected as the candidate key genes response to ammonia stress, and they would be validated in our next study with the ammonia-sensitive and ammonia-tolerant families of *L*. *vannamei*.

Besides the above key genes enriched in the significant GO terms and KEGG pathways, many other differentially expressed genes between LV_E and LV_C were potentially involved in immune and have been previously reported in penaeid shrimp [66–74], such as retinoblastoma-like protein (RBL), C-type lectin 2, hemocyanin, serine proteinase inhibitors, crustin, lysozyme, dual oxidase, prophenoloxidase-2, and peritrophin etc. (S3 Table). It was revealed that two WSSV paralogs interact with the conserved LxCxE motif of RBL of *L*. *vannamei* to sequester RBL and further to modulate host cell cycles and facilitate viral genome replication [75]. C-type lectins participate in the innate immunity to recognize and eliminate pathogens efficiently [76, 77], and hemocyanins have defense-related functions that are mediated through phenoloxidase activity [78, 79]. In addition, the expression of C-type lectins and hemocyanins could be significantly affected after virus challenge in the hepatopancreas of shrimp [80–86]. The serine proteinase inhibitors play a critical role in the regulation of proteinase-activated physiological and pathological process, such as blood coagulation, fibrinolysis, cellular remodeling, prohormone activation, complement system, and tumor metastasis, etc. [87], and they particularly protect cells from the rapid cytopathic effects of virus infection [88]. The previous study reported that the crustin protein exhibited antimicrobial activity against bacteria with strong inhibition [89], and lysozyme produced by shrimp hemocytes was found to play an important role in innate immunity in *L*. *vannamei* [90]. However, all of these genes were down regulated by ammonia stress in the shrimp (S3 Table). Xie et al. [91] reported that the envelope proteins of WSSV interact with peritrophin protein in the stomach when WSSV infecting the *L*. *vannamei*, so the up-regulated expression of peritrophin by ammonia stress would increase the susceptibility to pathogens. So we deduce that ammonia stress inhibit immune system activity and increase susceptibility to pathogens might be one of the main causes for the high mortality in deteriorating culture water for shrimp.

Generally, defense mechanisms are usually activated when organisms are exposed to environmental toxicants, among which cell death/apoptosis is usually triggered for defensing against stress or pathogen [92, 93]. However, some of the DEGs that were involved in the function of apoptosis made the shrimp failed to perform defensive function against ammonia stress, such as heat shock protein (Hsp) 70 and Hsp 90, etc. The apoptosis induced by stress proceeds through a defined biochemical process that involves Apaf-1 and caspase proteases, but Hsp 70 suppresses apoptosis by directly binding to Apaf-1 but not to procaspase-9, which could prevent recruitment of caspases to the apoptosome complex [94]. The expression of Hsp 70 was significantly up-regulated by ammonia stress, suggesting that damage resulting from ammonia stress is difficult to repair. Hsp 90s are chaperoned and make the proper folding of cellular proteins and help animals deal with the cellular protein damages under stress conditions [95], but its expression also was significantly down-regulated in by ammonia stress in the present study. In addition, the pathways involved in nitrogen metabolisms play an important role in reducing ammonia toxicity. Although the two pathways involved in nitrogen metabolisms (ko00250, q value = 0.933; ko00910, q value = 0.933) have not significantly changed by ammonia stress, but 8 genes in the two pathways were significantly changed (Table 5). The high activity of carbonic anhydrase (CA) facilitates cellular ammonia transport by providing H^+^ ions for the protonation of NH_3_, thus maintaining the *trans*-membrane NH_3_ gradient [96]. The un-ionized (NH_3_) state in water is the toxic form of ammonia to shrimp [7], so the significantly up-regulated expression of CA might reduce the toxicity in a certain degree to shrimp. The glutamate synthase (GLT1) catalyzes reactions that ammonia to glutamate, which is then used in a wide variety of biosynthetic reactions [97], so the significantly up-regulated expression of GLT1 in the present study would reduce ammonia toxicity to shrimp. The other four genes, D-aspartate oxidase (DDO), omega-amidase (NIT2), glutamate decarboxylase (GAD), and glutaminase kidney isoform (GLS), that involved in nitrogen metabolisms were significantly down regulated, which might reduce their ability to against ammonia toxicity according to the network diagram of the pathway (Fig 4G). Consequently, we deduce that the poor ability for reducing the ammonia toxicity and recovering the damages from ammonia stress might be another main cause for the high mortality in deteriorating culture water for shrimp. All of the results in the present study just reflect the gene and pathway responses in the early stage of acute ammonia stress, but the expression levels of the genes of the shrimp would be changed continuously under the ammonia stress (up or down). Therefore, we will perform the comparative transcriptome analysis for the later stage of acute ammonia stress to verify our deduction in the next study.

## Conclusion

The present study represents the first analysis for the identification of key genes and metabolic pathways response to ammonia stress at whole transcriptome level in shrimp. Our results revealed a total of 136 significantly differentially expressed genes that have strong homologies with known proteins in aquatic species, among which 94 genes were reported potentially related to immune function. Interestingly, among the 20 genes enriched in the significantly changed GO terms and KEGG pathways and have been reported in aquatic species, 16 genes potentially involved in the immune defense function and the expression changes of most of these genes would reduce the immune defense ability in shrimp. In addition, some of the significantly changed genes involved in apoptosis and nitrogen metabolisms that play an important role in reducing ammonia toxicity failed to perform the protection function. Because the ammonia stress could inhibit the immune system and increase the susceptibility of shrimp to pathogens, this study would provide important information for the future study for the molecular mechanism of immunosuppression resulting from ammonia stress in shrimp.

## Supporting Information

S1 Fig E-value and score distribution of unigenes matched to NR database. (a) E-value distribution of annotated unigenes. (b) Score distribution to annotated unigenes.
(TIF)

S2 Fig The distributions of the significantly changed GO terms of *Litopenaeus vannamei* under ammonia stress. (PNG)

S3 Fig The distributions of the significantly changed KEGG pathways of *Litopenaeus vannamei* under ammonia stress. (TIF)

S1 Table Primer sequences for the genes used for quantitative real time PCR. (DOC)

S2 Table The detail information of the functional annotation for the differentially expressed genes responds to ammonia stress in *Litopenaeus vannamei*. (XLS)

S3 Table List of the significantly differential expressed genes that have high homologies with known proteins in aquatic species. (XLSX)

S4 Table List of genes in significant enriched GO terms responded to ammonia stress in *Litopenaeus vannamei*. (XLSX)
