# Supplementary figures and images for: Transcriptome Analysis of the Hepatopancreas in the Pacific White Shrimp (Litopenaeus vannamei) under Acute Ammonia Stress
Source: PLoS One. 2016 Oct 19;11(10):e0164396. doi: 10.1371/journal.pone.0164396 (PMC5070816; doi:10.1371/journal.pone.0164396)

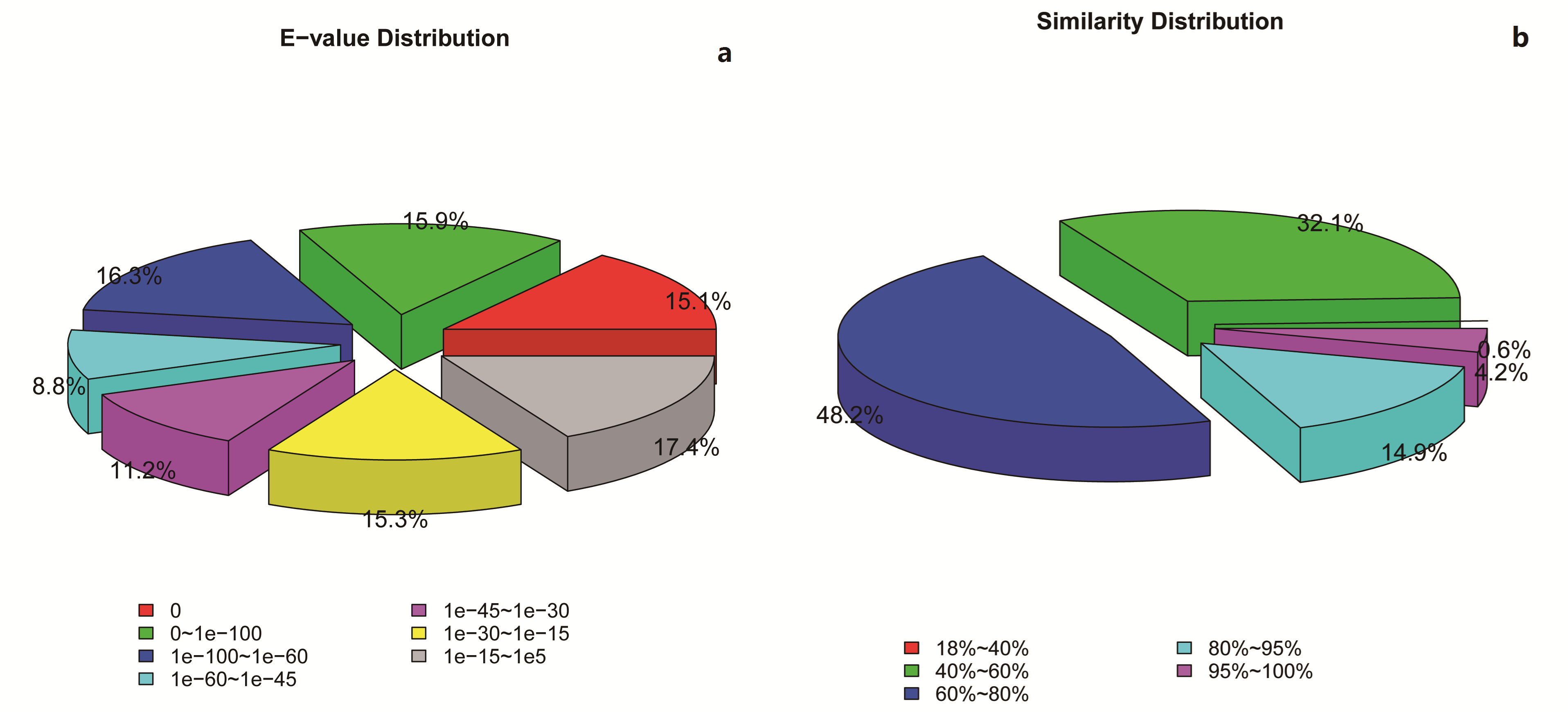

Supplement: S1 Fig — (a) E-value distribution of annotated unigenes. (b) Score distribution to annotated unigenes. (TIF) [file pone.0164396.s001.tif]

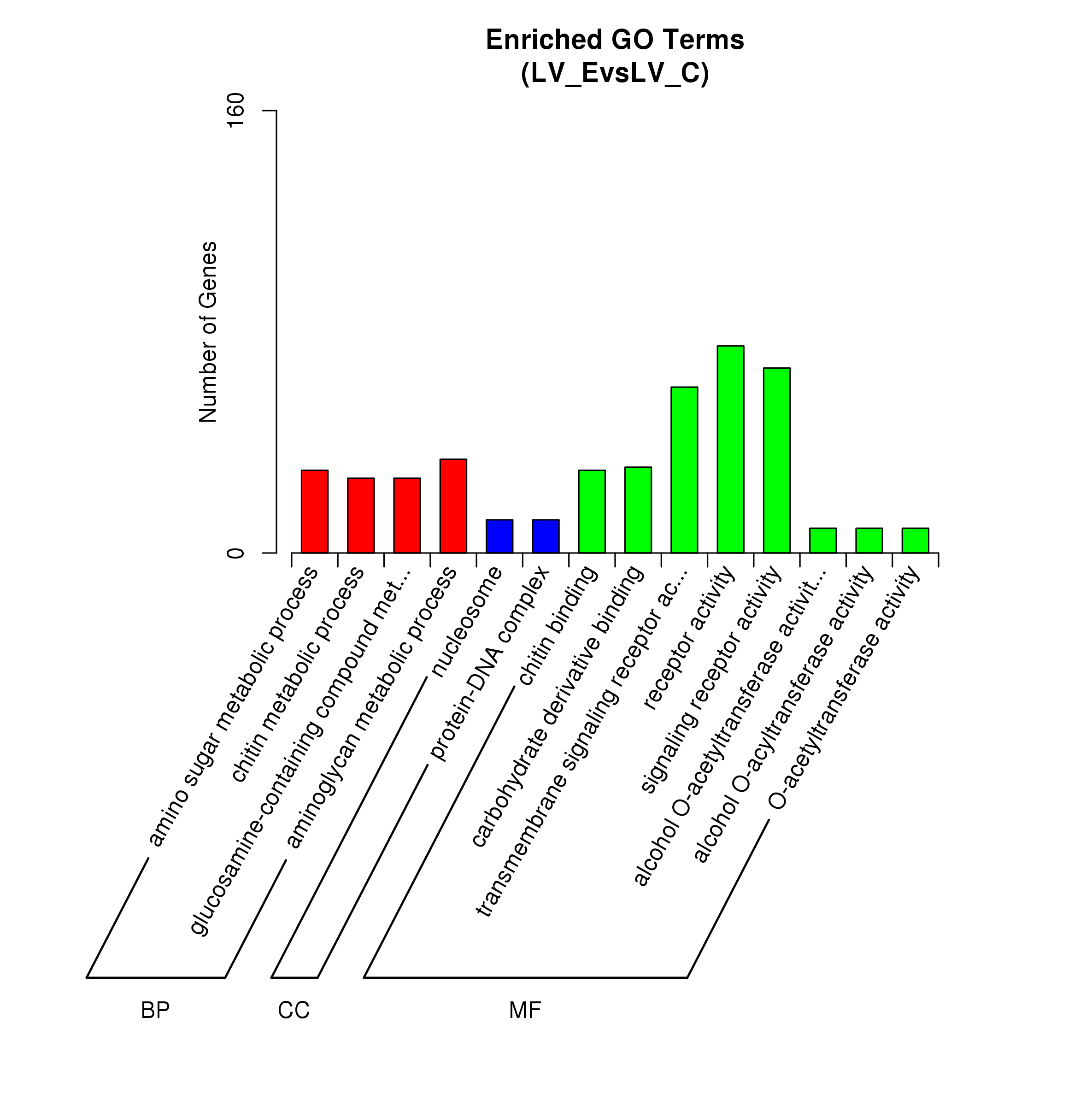

Supplement: S2 Fig — (PNG) [file pone.0164396.s002.png]

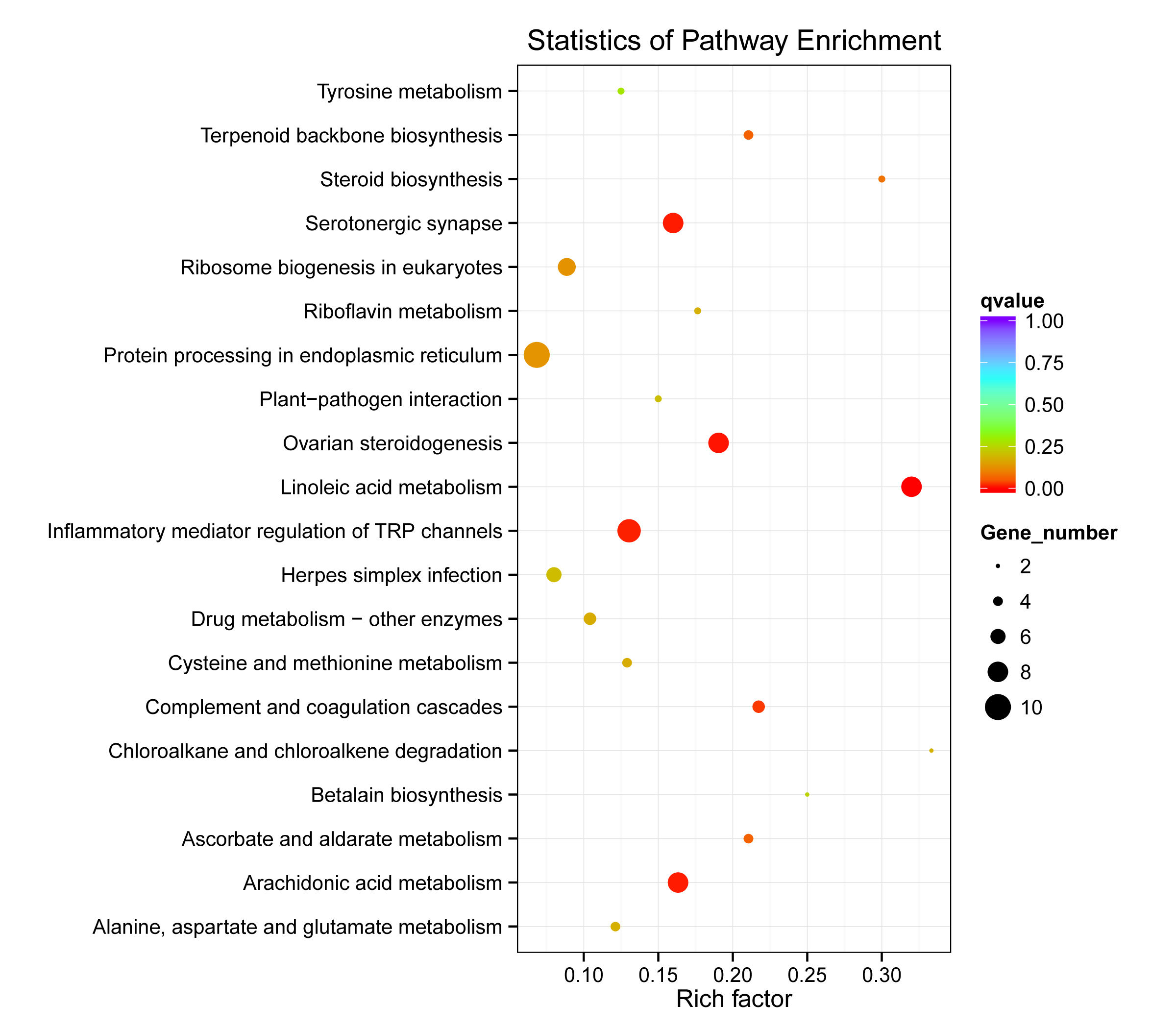

Supplement: S3 Fig — (TIF) [file pone.0164396.s003.tif]
