# Supplementary material for: Transcriptome Analysis of the Hepatopancreas in the Pacific White Shrimp (Litopenaeus vannamei) under Acute Ammonia Stress
Source: PLoS One. 2016 Oct 19;11(10):e0164396. doi: 10.1371/journal.pone.0164396 (PMC5070816; doi:10.1371/journal.pone.0164396)
Supplement: S1 Table — (DOC) [file pone.0164396.s004.doc]

S1 Table. Primer sequences for the genes used for quantitative RT-PCR

| **Gene name** | **Description** | **Primer sequences (5’-3’)** | |
| --- | --- | --- | --- |
|  |  | **Forward primers** | **Reverse primers** |
| CHH | molt inhibiting hormone-like protein | GCTCGACCGTCTCGCAAT | CTTCAGTAGGGTCGGCATAACC |
| nrf-6 | Nose resistant to fluoxetine protein 6 | GGTCGTAGAATCCCCTTGCA | GCTACGCTGGAAGAGCTTTCC |
| HSP70 | heat shock protein 70 | AGGAGTACGAGCATAAGATGAAGGA | AACCATGAATTTTGCCTCCAA |
| PvHm116 | putative antimicrobial peptide | TTGCTTCGACAGGTGTTTGG | GAAAACCTGCGATCCGAAGA |
| Duox | dual oxidase | CTGCGGCCGTTGTTGTTC | GCTACGCGACCGTCAAGTTC |
| ABCC1 | multidrug resistance protein 2 | TCTGCACCTGTTGATAGCCTGAT | GACATCGAGCCACAGAATTCTTC |
| RBL | retinoblastoma family-like protein (RBL) | GAGGTCACCCTCTTGAGCTG | CTTCCTAGCAGTGCTCCACC |
| Lysozyme | invertebrate-type lysozyme gene | GGGGAAGAGAAAAGAATGGC | TCGGTAGATCGAGGCTGTCT |
| SERPIN | serine proteinase inhibitor | TTCTTCTCGCCCTACAGCAT | ACTGGACCCTTTCCACCTCT |
| A2M | alpha-2-macroglobulin | ACCCGGCTGGTAGAGGTACT | GAGACCTGCTTCGAGGTGAC |
| PO | prophenoloxidase-2 | ACCCGGCTGGTAGAGGTACT | GAGACCTGCTTCGAGGTGAC |
| CASP2 | caspase | TGACGTCTTACACCTTTACCAAAGA | CGTCCTGTACAACTCTCCAAATTCT |
